# Syndecan-1 knockdown inhibits glioma cell proliferation and invasion by deregulating a c-src/FAK-associated signaling pathway

**DOI:** 10.18632/oncotarget.16733

**Published:** 2017-03-31

**Authors:** Shuang Shi, Dong Zhong, Yao Xiao, Bing Wang, Wentao Wang, Fu’an Zhang, Haoyang Huang

**Affiliations:** ^1^ Department of Neurosurgery, The 1st Affiliated Hospital of Chongqing Medical University, Chongqing 400016, China; ^2^ Experimental Research Center, The 1st Affiliated Hospital of Chongqing Medical University, Chongqing 400016, China

**Keywords:** syndecan-1, glioma, proliferation and invasion, c-Src/ FAK

## Abstract

Recent studies have shown that increased syndecan-1 (SDC1) expression in human glioma is associated with higher tumor grades and poor prognoses, but its oncogenic functions and the underlying molecular mechanisms remain unknown. Here, we examined SDC1 expression in datasets from The Cancer Genome Atlas and the National Center for Biotechnology Information Gene Expression Omnibus. Elevated SDC1 expression in glioma was closely associated with increases in tumor progression and shorter survival. We also examined SDC1 expression and evaluated the effects of stable SDC1 knockdown in glioma cell lines. SDC1 knockdown attenuated proliferation and invasion by glioma cells and markedly decreased PCNA and MMP-9 mRNA and protein expression. In a xenograft model, SDC1 knockdown suppressed the tumorigenic effects of U87 cells *in vivo*. SDC1 knockdown decreased phosphorylation of the c-Src/FAK complex and its downstream signaling molecules, Erk, Akt and p38 MAPK. These results suggest that SDC1 may be a novel therapeutic target in the treatment of glioma.

## INTRODUCTION

Glioblastoma multiforme (GBM), one of the most common primary brain tumors in adults, is highly aggressive and has a poor prognosis [1]. Despite extensive research, therapies for GBM have not improved much; consequently, patient survival has increased only slightly [2]. Although many studies have shown that various mechanisms are involved in the malignancy of glioma and its infiltration into other tissues, the regulatory factors involved remain largely unknown [3]. Additional investigations of the molecular mechanisms underlying the progression of human glioma are therefore urgently needed.

Syndecans are an evolutionarily conserved family of type I transmembrane proteins [4] which lack a common molecular structure and bind various ligands and receptors, including FGFs, vascular endothelial growth factors (VEGFs), transforming growth factor-β (TGF-β), platelet-derived growth factors (PDGFs), integrin, and VEGFR. They also regulate various signaling events both inside and outside cells; for example, syndecans affect the organization of the ECM and cytoskeleton by binding to phosphatidylinositol-4,5-bisphosphate and protein kinase Cα(PKCα), the activation of which leads to the formation of stress fibers and focal adhesions [5, 6]. By binding to other molecules, syndecans play dual roles as both cell adhesion receptors and docking receptors and are involved in multiple pathologic processes, including cancer cell proliferation and invasion, angiogenesis, host defense mechanisms, and matrix remodeling [7, 8]. Syndecan-1 (SDC1) is the most extensively studied member of the family. Studies indicate that SDC1 expression varies among cancer types and that differential expression in stromal compartments and carcinoma cells are strongly associated with tumor aggressiveness and clinical outcomes [9, 10]. In particular, SDC1 expression is elevated in glioma compared with normal brain tissues [11, 12], and Xu *et al*. found that SDC1 expression in human glioma correlates with advanced tumor progression and poor prognosis, indicating that SDC1 might play a pivotal role in the progression of glioma [13]. However, the specific mechanisms by which SDC1 promotes glioma remain unknown.

Recent studies have revealed that syndecans cooperate with integrins to regulate adhesion to a variety of ECM ligands, and this cooperation is required for focal adhesion formation and signal transduction [14]. One example of this cooperation is the close association between SDC1 and integrin [15]. Integrins are heterodimeric transmembrane receptors consisting of α and β subunits bound together noncovalently [16]. Integrins attach intracellular matrix receptors, such as the non-receptor tyrosine kinases focal adhesion kinase (FAK) and c-src, and other signaling proteins to the cytoskeleton; this recruitment and the subsequent activation of these proteins forms a focal adhesion [17]. Integrins also play a vital role in cell adhesion, migration, differentiation, and proliferation in tumors [18–20]. Previous studies have also shown that integrins play a vital role in glioma tumorigenesis [21] and that c-src and FAK are closely associated with key biological processes in glioma [22, 23].

In this study, we examined the functions of SDC1 and its relationship to the integrin-mediated signaling pathway in several human glioma cell lines. Our results demonstrate that knockdown of SDC1 expression inhibits glioma proliferation and invasion both *in vitro* and *in vivo*. Furthermore, SDC1 knockdown might inhibit integrin-mediated signaling by deregulating the c-src/FAK-associated signaling pathway and in turn attenuating the expression of PCNA and MMP-9.

## RESULTS

### Elevated SDC1 expression in glioma is closely associated with increased tumor grade and poor prognosis

**Figure 1:**
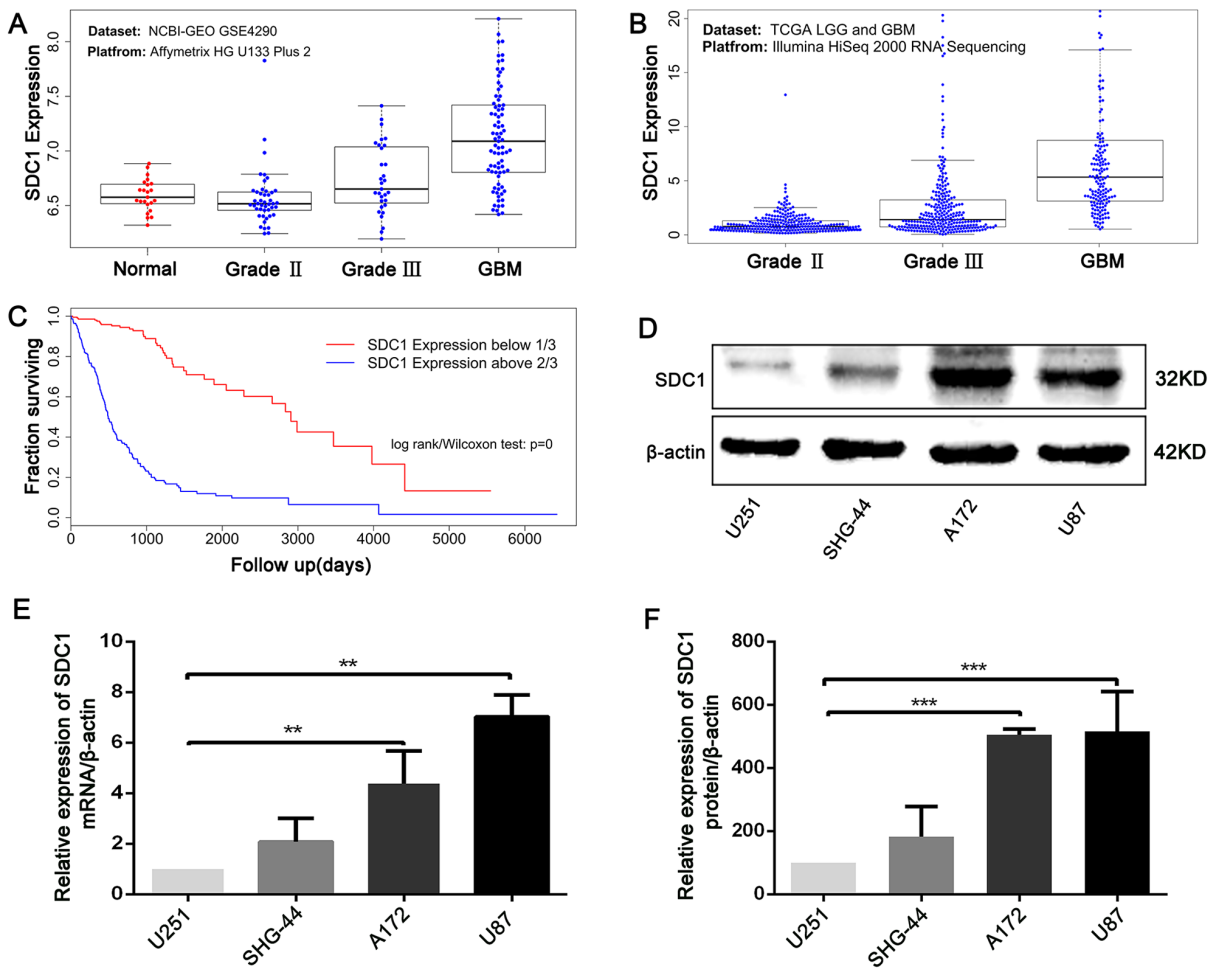
SDC1 is overexpressed in clinical glioma samples and glioma cell lines **(A)**, **(B)** SDC1 expression levels in glioma tumors with different grades from the NCBI-GEO GSE4290 and TCGA LGG and GBM datasets, *p*<0.0001. **(C)**. Kaplan–Meier survival curves in glioma patients according to SDC1 expression. High expression: SDC1 levels in the top third; Low expression: SDC1 levels in the bottom third, *p*=0. **(D)**, **(E)**, **(F)** SDC1 mRNA and protein expression in various glioma cell lines. Data are shown as the means ± SD of three independent experiments. **p<0.05, ***p<0.01.

To confirm the associations between elevated SDC1 expression in human glioma and increases in tumor progression and poor prognosis, combined data from TCGA for the LGG and GBM datasets and from the NCBI-GEO GSE4290 dataset were analyzed. Elevated SDC1 levels (Figure 1A and 1B) were correlated with more advanced tumor grades in both datasets (Kruskal-Wallis non-parametric test, *p*<0.0001 for both). Moreover, analysis of TCGA patient survival after segregation by SDC1 expression revealed that elevated SDC1 expression predicted prognosis (Figure 1C). Glioma patients with SDC1 levels in the top third had dramatically decreased survival compared to those with SDC1 levels in the lowest third (Kaplan–Meier survival analysis, *p*=0 in both log-rank and Wilcoxon tests). These data suggest that elevated SDC1 expression plays a crucial role in the tumorigenesis of glioma.

### Establishment of stable SDC1-knockdown A172 and U87 cell lines

**Figure 2:**
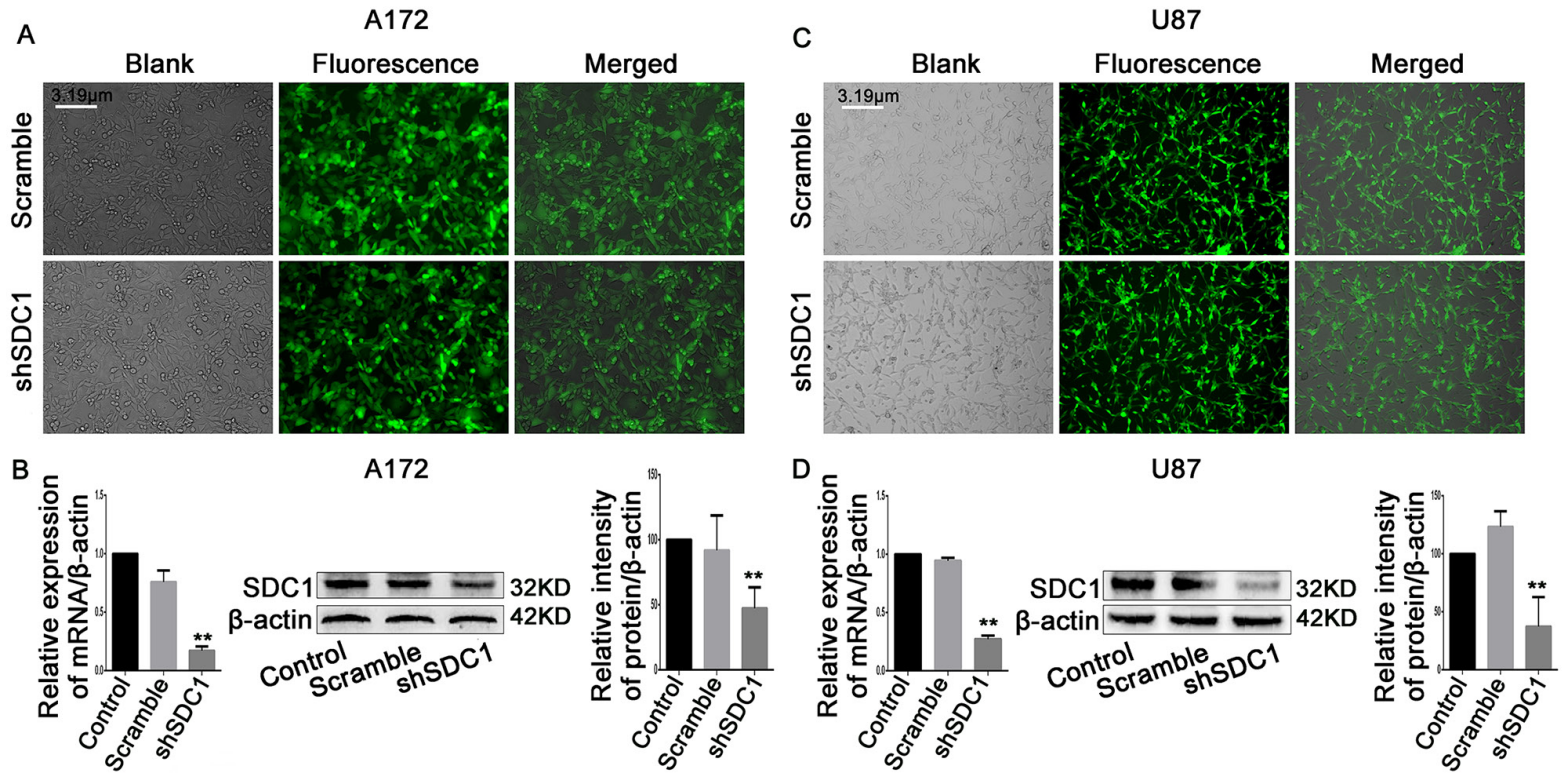
SDC1 knockdown inhibits the expression of PCNA and MMP-9 mRNA and protein **(A)**, **(B)** Green fluorescent protein (GFP) expressed in A172 and U87 cells transfected with scramble or SDC1 shRNA (200×). **(C)**, **(D)** qRT-PCR and Western blots revealed that SDC1, PCNA, and MMP-9 expression decreased in the shSDC1 group compared to the scramble group. Data are shown as the means ± SD of three independent experiments. **p<0.01.

Next, we analyzed SDC1 expression in different glioma cell lines. qRT-PCR and Western blots revealed that SDC1 expression was higher in U87 and A172 cells than in U251 and SHG-44 cells (Figure 1D, 1E, and 1F, **p<0.05, ***p<0.01). To determine the effects of SDC1 expression knockdown in glioma cells, A172 and U87 cells were transfected with lentiviral vector carrying SDC1 shRNA. The shSDC1 group consisted of cells with stable SDC1 knockdown, the control group consisted of untransfected cell lines, and the scrambled group consisted of cell lines transfected with scrambled shRNA. Fluorescent staining confirmed that transfected cells expressed green fluorescent protein and indicated high transfection efficiency after puromycin selection (Figure 2A and 2B). qRT-PCR showed that SDC1 mRNA levels in the shSDC1 group decreased by 82.9% and 72.82% in A172 and U87 cells, respectively, compared to the control group; similarly, Western blots demonstrated that SDC1 protein levels in the shSDC1 group decreased by 52.71% and 62.73% in A172 and U87 cells, respectively (Figure 2C and 2D, **p<0.01). Taken together, these data indicate that stable shRNA-mediated knockdown of SDC1 expression in A172 and U87 cells was successful.

### SDC1 knockdown suppresses cell proliferation and colony formation in A172 and U87 cells

**Figure 3:**
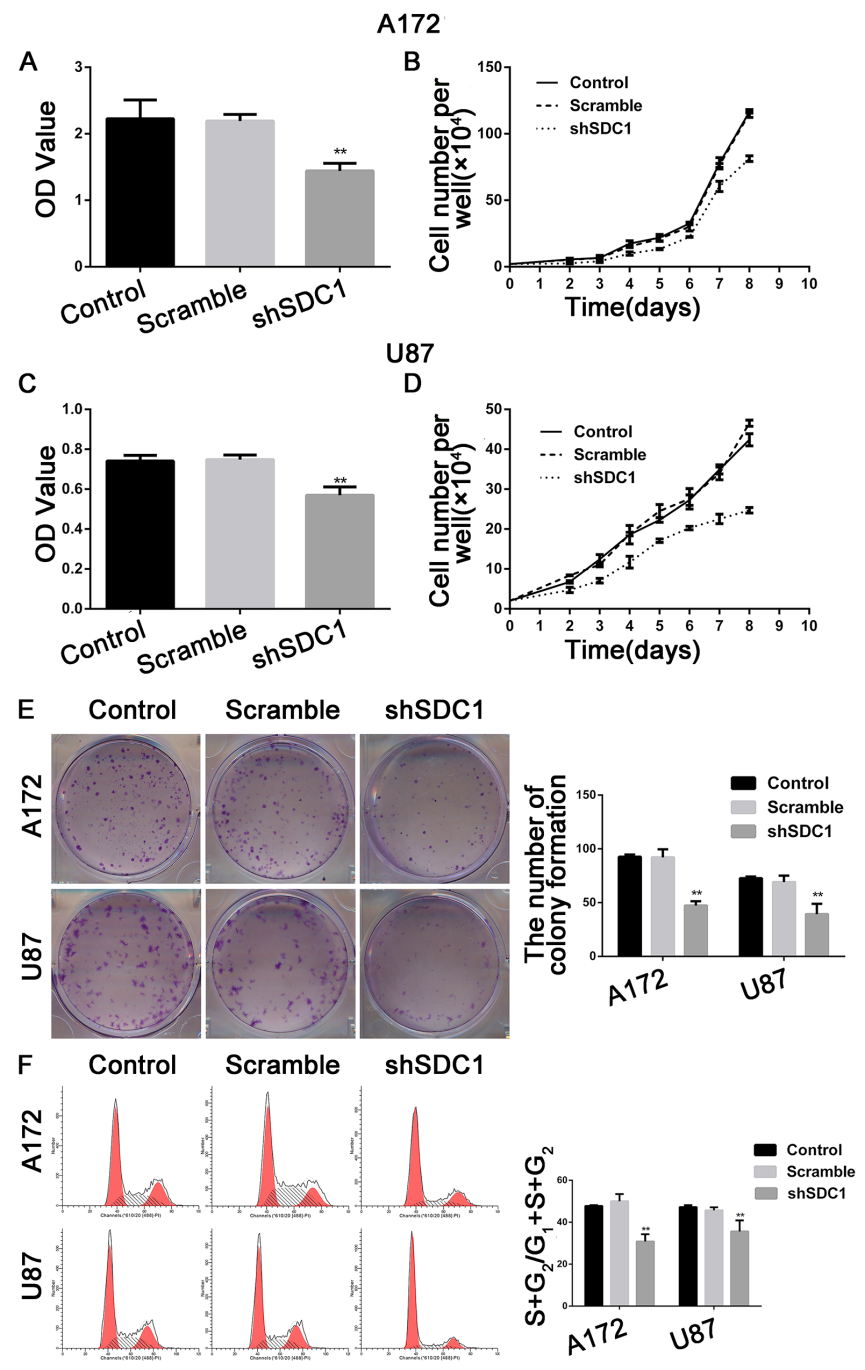
SDC1 knockdown inhibits the proliferation of A172 and U87 cells *in vitro* **(A)**, **(C)** MTT incorporation assay for A172 and U87 cells at 72 hours. **(B)**, **(D)**. Growth curves for A172 and U87 cells. **(E)** SDC1 knockdown suppressed colony formation in A172 and U87 cells compared to the control group. **(F)** SDC1 knockdown decreased the (S+G2)/(G1+S+G2) proliferation index in the shSDC1 group in A172 and U87 cells. Data are shown as the means ± SD of three independent experiments. *p<0.05, **p<0.01.

The MTT assay showed that the viability of shSDC1 group A172 and U87 cells decreased after 72h of culture compared to control and scramble group cells (Figure 3A and 3C, *p<0.05, **p<0.01), and the growth curve showed a time-dependent delay in growth in the shSDC1 group (Figure 3B and 3D, *p<0.05). In addition, colony formation assays revealed that cell proliferation and colony formation were inhibited in SDC1-knockdown cells (Figure 3E, **p<0.01). These results indicated that SDC1 knockdown suppressed proliferation and colony formation in A172 and U87 cells.

### SDC1 knockdown inhibits cell cycle progression in A172 and U87 cells

**Figure 5:**
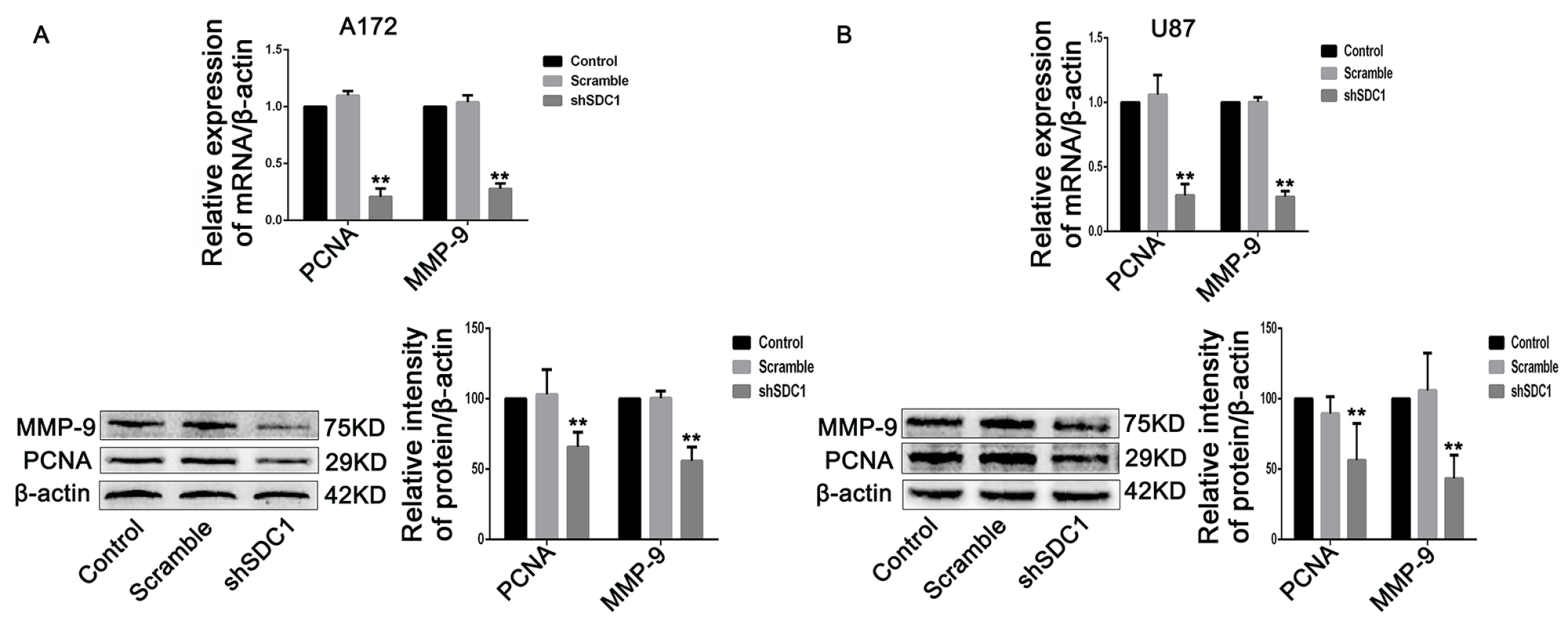
SDC1 knockdown inhibits the expression of PCNA and MMP-9 mRNA and protein **(A)**, **(B)** qRT-PCR and Western blots showed that PCNA and MMP-9 expression decreased in the shSDC1 group compared to the scramble group. Data are shown as the means ± SD of three independent experiments. **p<0.01.

The effects of SDC1 knockdown on cell cycle progression in A172 and U87 cells were analyzed using a flow cytometer. The number of cells in the S phase decreased, and G0/G1 phase arrest increased, in the shSDC1 group. The proliferation indices ((S+G2)/(G1+S+G2)) of the shSDC1 groups (30.84±3.41% and 35.64±5.24%) decreased compared to the control (47.76±0.39% and 47.20±0.92%) and scramble groups (50.02±3.39% and 45.69±1.44%) in both A172 and U87 cells, respectively (Figure 3F, **p<0.01). We then confirmed the decrease in S phase population using qRT-PCR, which revealed that PCNA mRNA levels in the shSDC1 groups decreased by 79.33% and 72.08% in A172 and U87 cells, respectively, compared to the control group. Western blots also showed that PCNA protein levels in the shSDC1 group decreased by 34.17% and 43.74% in A172 and U87 cells, respectively (Figure 5A and 5B, **p<0.01). These results confirmed that SDC1 knockdown decreased proliferation in A172 and U87 cells by inhibiting the S phase.

### SDC1 knockdown reduces migration and invasion in A172 and U87 cells

**Figure 4:**
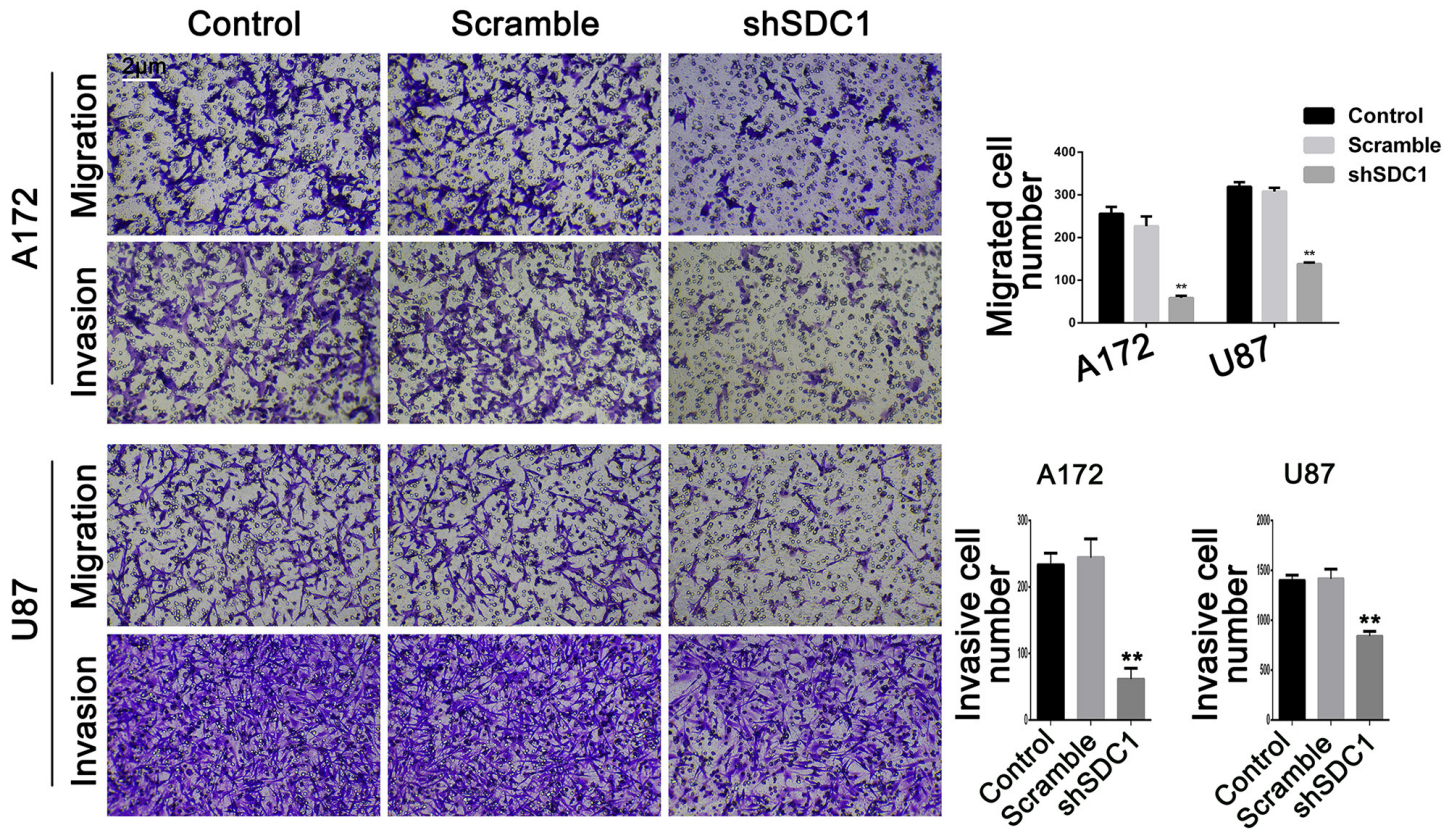
SDC1 knockdown decreases migration and invasion of A172 and U87 cells The effects of SDC1 knockdown on migration and invasion of A172 and U87 cells were assessed by Transwell assay. Data are shown as the means ± SD of three independent experiments. **p<0.01 (200×).

In the transwell migration experiment, SDC1 knockdown decreased cell migration compared to control and scramble groups in both A172 and U87 cells (58.40 ± 5.24 vs 255.8 ± 16.09 and 226.5 ± 22.84 in A172 and 138.1 ± 3.21 vs 319.0 ± 10.91 and 307.8 ± 8.83 in U87 cells, respectively, Figure 4, **p<0.01). Transwell chambers coated with Matrigel were then used to evaluate invasive ability. Cell invasion also decreased in both shSDC1 groups compared to the negative and blank control groups (61.67±16.26 vs 233.7±17.24 and 244.3±28.15 in A172 and 840.7±48.64 vs 1400±53.84 and 1416±98.33 in U87 cells, respectively, Figure 4, **p<0.01). Consistent with this result, qRT-PCR revealed that MMP-9 mRNA levels in the shSDC1 groups decreased by 72.82% and 73.15% in A172 and U87 cells, respectively, compared to the control groups, and Western blots revealed that MMP-9 protein levels in shSDC1 groups decreased by 54.82% and 56.65% in A172 and U87 cells, respectively (Figure 5A and 5B, **p<0.01). In summary, knockdown of SDC1 expression inhibited the invasive ability of glioma cells.

### SDC1 knockdown inhibits cell proliferation and invasion by deregulating c-src/FAK complexes in glioma cells

**Figure 6:**
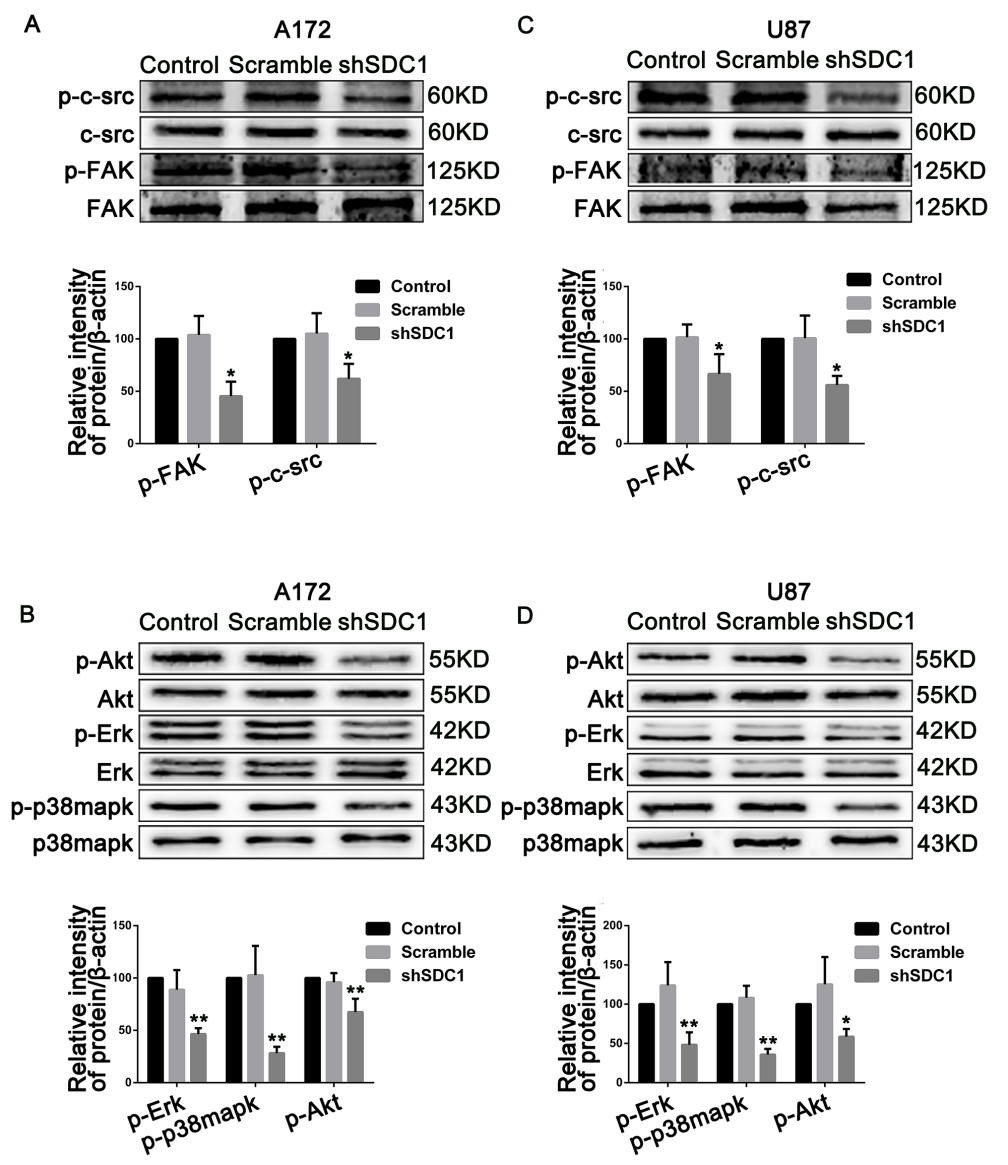
SDC1 knockdown suppresses phosphorylation of c-src/FAK and associated signaling pathway molecules in A172 and U87 cells **(A)**, **(B)** SDC1 knockdown attenuated phosphorylation of Tyr 416 in c-src and Tyr 397 in FAK in A172 and U87 cells. **(C)**, **(D)** Phosphorylation of Erk, p-38mapk, and Akt also decreased after SDC1 knockdown in A172 and U87 cells. Data are shown as the means ± SD of three independent experiments. *p<0.05, **p<0.01.

To explore the mechanisms underlying the SDC1 knockdown-induced inhibition of progression in glioma, we examined the activation of c-src/FAK complexes, which are important for focal adhesion in glioma cells. Compared to the control groups, phosphorylation of Tyr 416 in c-src and of Tyr 397 in FAK in the shSDC1 groups decreased 1.61- and 2.21-fold in A172 and 1.78- and 1.5-fold in U87 cells, respectively (Figure 6A and 6C, *p<0.05, **p<0.01). To further confirm that SDC1 knockdown deregulated phosphorylation of c-src/FAK complexes, activation of downstream signaling molecules closely associated with proliferation and invasion was examined. As expected, phosphorylation of Erk, p-38mapk, and Akt was suppressed 2.16-, 3.55-, and 1.48-fold in A172 and 2.07-, 2.80-, and 1.71-fold in U87 cells, respectively (Figure 6B and 6D, *p<0.05, **p<0.01). Based on corresponding total protein levels and the internal control, there were no detectable differences between the control and scramble groups. These results suggest that SDC1 silencing suppressed cell proliferation and invasion in A172 and U87 cells by deregulating the phosphorylation of c-src/FAK complexes and downstream signaling molecules.

### SDC1 knockdown inhibits tumor growth in subcutaneous xenografts of U87 glioblastoma cells

**Figure 7:**
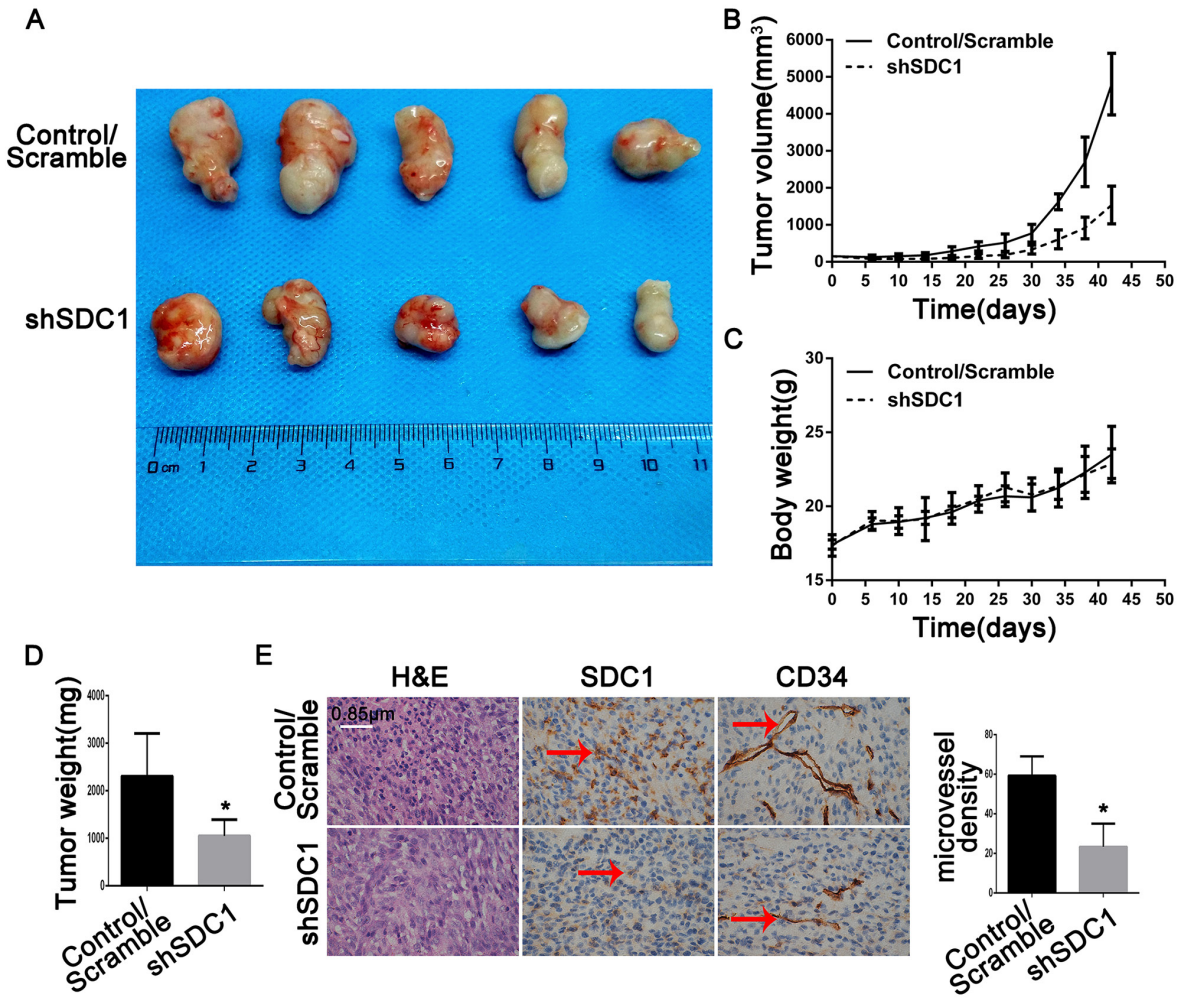
SDC1 knockdown inhibits growth and angiogenesis in subcutaneous tumor xenografts in nude mice **(A)** Forty-two days after subcutaneous injections of U87 cells transfected with scramble or SDC1 shRNA into the dorsal flanks of female nude mice, tumors were removed at necropsy and photographed. **(B)**. Growth of xenograft-induced tumors was measured externally twice a week over a 6-week period; tumor volumes were calculated and mean volumes ± SD are shown (n = 5). **(C)**. Mice were weighed twice a week; mean body weights ± SD are shown (n = 5). **(D)** Weights of tumors excised on day 42 were measured and means ± SD are shown (n = 5), *p<0.05. **(E)**. Sections of xenograft-induced solid tumors were immunostained with anti-SDC1 or anti-CD34 antibodies or with nonimmune serum (N/S) followed by counterstaining with hematoxylin (400×).

A tumor growth curve showed that tumors derived from shSDC1 group cells grew more slowly than those derived from control/scramble group cells. Six weeks after injection, the tumor volumes in the control/scramble and shSDC1 groups were 4800 ± 1530 mm^3^ and 830 ± 500 mm^3^, respectively (Figure 7D; *p<0.05). Mean tumor weight in the shSDC1 group was similarly reduced compared to the control/scramble group (0.89 ± 0.34 vs 2.30 ± 1.05 g, Figure 7A and 7B; *p<0.05). Tumor burdens and body weights were also measured. However, no significant differences in body weight were detected between the two groups of mice (Figure 7C, *p>0.05), suggesting that tumor formation did not lead to increases in tumor burden within 42 days of xenograft. Postmortem tumor tissues were then collected and sectioned for immunohistochemical staining. SDC1 expression was lower in the shSDC1 group than in the control/scramble group. Additionally, CD34 expression was higher in the control/scramble group than in the shSDC1group, suggesting that SDC1 plays a role in GBM angiogenesis (Figure 7E). These results indicate that SDC1 might play a critical role in glioma proliferation and regulate angiogenesis in GBM.

## DISCUSSION

In this study, SDC1 expression was analyzed in TCGA glioma samples, which included both low-grade glioma (LGG) and GBM datasets, and in the NCBI-GEO GSE4290 dataset [24]. SDC1 expression did not differ between Grade II tumor tissues and normal tissues in the GSE42900 dataset, but was higher in Grade III and GBM tumor tissues compared to normal tissues. Results for both the TCGA and GSE4290 datasets indicated that elevated SDC1 expression is closely associated with increased progression in glioma tumors; the comparatively low malignancy of Grade II glioma might partially explain the lack of an increase in SDC1 expression in that stage. Interestingly, the clinical data in TCGA showed that glioma patients with elevated SDC1 expression did not survive as long as patients with lower SDC1 expression (Figure 1A–1C). Taken together, these results indicate that SDC1 plays a crucial role in the tumorigenesis of glioma.

To confirm that finding, we examined the effects of SDC1 knockdown on glioma cell growth. Our results revealed that SDC1 knockdown only weakly inhibited glioma cell growth in MTT assays and at the beginning of the growth curve. However, SDC1 knockdown markedly inhibited growth in both A172 and U87 cells after 9 and 12 days in colony formation assays. It is possible that these differences might result from differences between the assays in the microenvironment during cell growth. Growth curves and colony formation as measured in the MTT assay reflect the growth of a relatively small number of cells. At early time points in this assay, an unfavorable microenvironment might have slowed the growth of the few cells that were initially present. However, as the number of cells began to increase, the microenvironment might have become more favorable for cell growth, thereby accelerating the proliferation of glioma cells. In addition, we found that U87 cells had stronger migration ability and much stronger invasion ability than A172 cells. This finding might be due to an increased sensitivity to Matrigel, which might enhance invasive ability, in U87 cells compared to A172 cells. PCNA, the expression of which is elevated in proliferating cells and in most malignant tumor cells, is commonly used as a proliferative/malignancy biomarker in cancer [25]. In addition, expression of MMP-9, which is associated with cell migration and extracellular matrix degradation, reflects the migration and invasion of tumor cells [26]. Our data suggest that SDC1 knockdown inhibits the proliferation and invasion of A172 and U87 cells by inhibiting PCNA and MMP-9 expression.

We also determined the effects of SDC1 knockdown on tumor growth *in vivo* using a xenograft model. Tumors resulting from the injection of shSDC1 cells grew more slowly than those resulting from control cells (Figure 6A, 6B, and 6D), as evidenced by both lower tumor weights and volumes. This result was consistent with our *in vitro* data and confirmed that SDC1 knockdown also inhibited glioma cell growth *in vivo*. In addition, we examined SDC1 expression in cancer cells using immunohistochemistry and found that SDC1 expression was reduced in the SDC1 knockdown group compared to the control/scramble group (Figure 6E). These results further confirm that SDC1 might be a key signaling molecule in the regulation of glioma cell proliferation.

Upon integrin recruitment, FAK binds to c-src to form a dual-kinase complex at the sites of cell–substratum focal adhesions [17], and these c-src/FAK complexes coordinate cell behavior by regulating multiple downstream pathways and molecules, including Akt, p38-mapk, and Erk kinase [27, 28]. Autophosphorylation of c-src/FAK complexes at either Tyr 397 in FAK or Tyr 416 in c-src is a critical event in integrin-mediated signaling. Notably, pY397FAK binds the SH2 domains of SFKs with high affinity, and this interaction up-regulates c-src kinase activity and, in turn, the phosphorylation of Tyr 416 in c-src. Activated c-src binds to pY397FAK and then phosphorylates additional sites on FAK to form c-src/FAK complexes [24]. In our study, SDC1 knockdown was also accompanied by a decrease in c-src Tyr 416 and FAK Tyr 397 phosphorylation, which is indicative of the disassembly of c-src/FAK complexes and is consistent with decreased proliferation and invasion (Figure 5A and 5B). Concomitantly, phosphorylation of Erk, p38mapk, and Akt, which are involved in pathways downstream of c-src/FAK complexes and are closely associated with proliferation and invasion, also decreased (Figure 5C and 5D). Because glioma cells express predominantly the α_v_and β_1_ subunits of integrin [29] and SDC1 can interact with bothof these submits [14], SDC1 knockdown might inhibit integrin-mediated signaling by deregulating c-src/FAK-associated signaling pathways in glioma cells.

Recent studies have demonstrated that angiogenic endothelial glioma tumor cells express integrin α_v_β_3_/α_v_β_5_ [30, 31], which plays a role in angiogenesis. Drugs that target integrin α_v_β_3_/α_v_β_5_ and its ligands, including cilengitide, vitaxin, and CNTO 95 [32], have been evaluated in recent clinical trials. However, phase III trials show that Cilengitide, which specifically binds to integrin α_v_β_3_/α_v_β_5_ receptors, in combination with temozolomide failed to improve survival in GBM patients [33, 34]. Moreover, integrin α_v_β_3_-c-src complexes promote anchorage-independence and tumor progression [35], implying that integrin may play inconsistent roles in transducing extracellular signals after binding to different ligands. In addition, SDC1 regulates the activation of integrin α_v_β_3_/α_v_β_5_ via its extracellular domain [15]. Together, these results suggest that SDC1 plays a vital role in the angiogenesis of glioma. Here, we detected a decrease in microvessel density as indicated by CD34, which is the most reproducible marker of endothelial cells in intratumoral microvessels, in the *in vivo* shSDC1 group (Figure 6E). However, the mechanism by which SDC1 knockdown inhibits angiogenesis in glioma remains unknown. Interestingly, recent studies indicate that SDC1 can be transferred between cells via exosomal release [36, 37]. We hypothesize that SDC1 might be transferred from glioma cells to vascular cells via exosomal release and thereby regulate the activation of integrin α_v_β_3_/α_v_β_5_ on vascular endothelial cells. Our current results might therefore support a new mechanism by which SDC1 contributes to the angiogenesis of glioma.

In summary, we found that high SDC1 expression in human glioma was strongly associated with more advanced tumor stages and shorter survival. Furthermore, SDC1 knockdown inhibited the proliferation and invasion of human glioma cells at least in part by inhibiting integrin-mediated signaling via deregulation of the c-src/FAK-associated signaling pathway. Together, these results indicate that SDC1 might be a promising prognostic predictor and a novel therapeutic target in the treatment of GBM. Additional studies are needed to further characterize the specific mechanisms by which SDC1 regulates proliferation and invasion in glioma.

## MATERIALS AND METHODS

### Materials and reagents

The U251, SHG-44, A172, and U87 human glioma cell lines were purchased from the China Center for Type Culture Collection (Shanghai, China). The lentiviral vectors, which generated small hairpin RNA (shRNA) targeting SDC1 or scramble shRNA, were constructed by and purchased from Shanghai Genechem Co. The transwell chamber was purchased from Millipore Corporation (MA, USA). 3-(4,5-dimethyl-2-thiazolyl)-2,5-diphenyl-2-H-tetrazolium bromide (MTT) was obtained from Genview Corporation (Shanghai, China). Matrigel was purchased from BD Biosciences (MA, USA). The SDC1 antibody was obtained from Thermo Fisher Scientific (MA, USA), and the PCNA, MMP-9, and β-actin antibodies were purchased from Abcam (MA, USA). Antibodies against total or phosphorylated FAK, src, Akt, Erk1/2, and p38MAPK were purchased from Cell Signaling Technology (MA, USA). Primers for β-actin, SDC1, PCNA, and MMP-9 were purchased from TaKaRa Biotechnology (Shiga, Japan).

The BALB/c-nu nude mice (females, 4-6 weeks old) were purchased from and housed and fed in the Animal Center of Chongqing Medical University under standard conditions according to the Institute's guidelines. All animal experiments were approved by the Animal Ethics Committee of Chongqing Medical University.

### Cell culture

U251, SHG-44, A172, and U87 cells were cultured in DMEM medium supplemented with 10% FBS (HyClone, UT, USA) and 1% penicillin-streptomycin (Beyotime Biotechnology, Jiangsu, China). Cells were maintained at 37°C in a humidified incubator with 5% CO_2_. When cells reached 80-90% confluence (usually in 2 or 3 days), they were harvested using trypsin (0.25%) with 0.01% ethylenediamine tetraacetic acid (EDTA) and seeded (1:2) into new culture flasks with complete DMEM. The media were replaced every day.

### Quantitative real-time polymerase chain reaction

Total RNA was extracted from different cell lines using Trizol reagent (TaKaRa Biotechnology, Shiga, Japan) per the manufacturer's instructions. Complementary DNA (5 µg) was synthesized by reverse-transcribing total RNA using a PrimeScript™ RT reagent kit with gDNA Eraser (TaKaRa Biotechnology, Shiga, Japan) according to the manufacturer's protocol. Equal amounts of cDNA samples were used as templates for real-time PCR to measure mRNA levels. qRT-PCR was performed using a CFX96 Real-Time PCR system (Bio-Rad, CA, USA) and a SYBR Premix Ex Taq™ II PCR Kit (TaKaRa Biotechnology, Shiga, Japan); β-actin was used as an internal control. The primers were as follows: β-actin: 5’-CATGTACGTTGCTATCCAGGC-3’ (sense) and 5’-CTCCTTAATGTCACGCACGAT-3’ (antisense); syndecan-1: 5’-CGTGGGGCTCAT CTTTGCT-3’ (sense) and 5’-TGGCTTGTTTCGGCTCCTC-3’ (antisense); PCNA: 5’-GTAATGTCGATAAAGAGGAGGAAGC-3’ (sense) and 5’-CATACTGAGTGTCA CCGTTGAAGAG-3’ (antisense); MMP-9: 5’-TGTACCGCTATGGTTACACTCG-3’ (sense) and 5’-GGCAGGGACAGTTGCTTCT-3’ (antisense). The PCR amplification consisted of 40 cycles of 95°C for 5s and 58°C for 30s after an initial denaturation step of 95°C for 30s, and the results were collected and analyzed (Standard Curve Method) using the Bio-Rad CFX Manager, version 3.1 according to the manufacturer's instructions. The experiments were performed in triplicate.

### Lentivirus transfection assays

To inhibit SDC1 expression, the oligo DNA with the highest interference efficiency as identified via qRT-PCR in a preliminary experiment was used to infect glioma cells. The SDC1 shRNA sequence was 5’-GACTGCTTTGGACCTAAAT-3’, and the scramble shRNA sequence was 5’-TTCTCCGAACGTGTCACGT-3’. To establish stable SDC1-knockdown cell lines, the cells were seeded into 6-well plates 12 hours prior to transfection. After they reached 30% confluence, the cells were incubated with lentiviral vectors in serum-free transfection medium (SIGMA). The cells were then transferred to fresh complete DMEM after 24 hours and cultured for an additional 72 hours. Stably-transfected cells were isolated using puromycin selection. Cell proliferation and invasion were then examined in cells with stable SDC1-knockdown.

### Cell viability and cell count assays

Cell viability assays were performed to observe and compare proliferation ability in different cells. The cells were plated in 96-well plates at a density of 1×10^4^ per 200 μL. After incubating for 72 hours, 20 μL of MTT solution (5 mg/mL, Genview Co.) was added into each well followed by 4 additional hours of incubation. The culture medium was then removed and 150 mL of DMSO was added to solubilize the crystals for 10 min at room temperature. The absorbance at 570 nm was read using a Microplate reader (M88, Thermo). For the cell count assay, cells were seeded at a density of 2×10^4^ cells/well in 6-well plates and removed by trypsinization every 24 hours. The number of viable cells per well was counted after trypan blue staining using a hemocytometer. All measurements were performed independently in triplicate.

### Colony formation assay

Five hundred cells per group were seeded into 6-well plates and cultured for 9-12 days. When macroscopic colonies in the plate were visible to the naked eye, the culture was stopped and surviving colonies were fixed with paraformaldehyde for 30 minutes and then stained with 0.5% crystal violet for 15-20 minutes. Colonies that were more than 50 mm diameter were counted directly on the plate. Statistical analysis was performed using data from at least three independent experiments.

### Cell cycle analysis

For cell cycle analysis, 2×10^5^ cells per group were seeded into six-well culture plates, incubated with serum-free medium for 16 hours, and then cultured for an additional 72 hours with complete DMEM at 37°C. The cells were then harvested using 0.25% Trypsin digestion, washed in ice-cold PBS twice, and fixed with cold 70% ethanol at 4°C overnight. After they were washed to remove the ethanol, the cells were treated with 0.01% RNase (10 mg/mL; Sigma) at 37°C for 10 minutes and then stained with 0.05% propidium iodide at 4°C in dark for 20 minutes. Cell cycle distribution was determined using a FACScan (BD Influx) and analyzed using Modfit software (Phoenix). Each sample was independently measured at least three times.

### Transwell assays for migration and invasion

The migration assays were performed using a 24-well Transwell chamber (Millipore, Millicell, USA) with membranes (pore size, 8 µm). 7×10^4^ cells in 200 μL serum-free DMEM were seeded into the upper chambers and 600 μL of 10% FBS in DMEM, which served as a chemotactic agent, was added to the lower chamber. U87 and A172 cells were incubated in the chambers at 37°C for 7 and 20 hours, respectively, and nonmigratory cells in the upper chambers were then removed from the membranes. The migrated cells remaining on the bottom surfaces of the membranes were fixed in 600 μL 4% paraformaldehyde for 30 minutes at room temperature, stained with 600 μL 0.1% crystal violet dissolved in methanol for 10 minutes, and then washed 3 times in PBS. Numbers of migrated cells were then counted using an inverted microscope (Leica, × 200). Chambers in which the upper membranes had been precoated with Matrigel (40 μL, BD Biosciences) were used for invasion assays. All assays were performed in triplicate for each sample and 5 microscopic fields were counted per insert.

### Western blotting analysis

Total protein was isolated from glioma cells using SDS Buffer (Beyotime Biotechnology, China) and the BCA Protein Assay Kit (Beyotime Biotechnology, China) was used to measure protein concentration according to manufacturer's instructions. Subsequently, 10-40 µg of total protein per group were separated on 10% sodium dodecyl sulfate polyacrylamide gels and transferred onto polyvinylidene fluoride membranes (Millipore, Billerica, MA, USA). After they were blocked in 5% nonfat milk, the membranes were incubated with the following primary antibodies: anti-FAK, anti-phospho-FAK Tyr397, anti-Src, anti-phospho-Src Tyr416, ERK1/2, anti-phospho-ERK1/2 (Thr202/Tyr204), anti-p38MAPK, anti-phosphor-p38MAPK (Thr180/Tyr182), anti-Akt, and anti-phospho-Akt Ser473 from Cell Signaling Technology; anti-Syndecan-1 from Thermo Fisher Scientific; anti-PCNA and anti-MMP-9 from Abcam. After washing in TBST for 30 min, the corresponding HRP-conjugated secondary antibodies were added and bands were visualized using the ECL chemiluminescence kit (Beyotime Biotechnology, China). β-actin was used as the internal control. All experiments were independently repeated 3 times. Densitometric analyses of Western immunoblots were performed using a Fusion FX7 (Vilber Lourmat, France) equipped with FUSION-CAPT analysis software. Quantitative densitometric analysis of the Western blots normalized to β-actin densitometric units are presented. Values for scramble and shSDC1 groups compared to controls are plotted as means ± SEM (indicated by error bars).

### Xenograft models and tumor formation

All animal experiments were approved by the Animal Ethics Committee of Chongqing Medical University. Ten BALB/c-nu nude mice (females, 4-6 weeks old) were purchased from and housed and fed in the Animal Center of Chongqing Medical University under standard conditions according to the Institute's guidelines. The mice were randomly divided into two groups (n=5). To construct a subcutaneous xenograft model [38], SDC1-knockdown U87 cells or corresponding scramble-transfected cells were injected at 5×10^5^ cells/150 μL phosphate-buffered saline subcutaneously into the dorsal flank (n = 5 mice/group) as described previously; mice were then monitored for 4-6 weeks. Tumor volumes were measured twice a week with a caliper and calculated using the following formula: π/6×larger diameter×(smaller diameter)^2^. The mice were then sacrificed and tumors were resected, weighed, and preserved for hematoxylin and eosin (H & E) and immunohistochemical staining. The data are presented as means ± SD.

### Immunohistochemistry

Immunohistochemistry was performed to measure SDC1 and CD34 expression. Portions of the tumor tissues were fixed with 4% paraformaldehyde and embedded with paraffin using standard methods. The tissue sections were cut into 4 μm-thick sections, deparaffinized in xylene, and rehydrated with graded ethanol. After neutralization of endogenous peroxidase and antigen retrieval, slides were first incubated with anti-SDC1 and -CD34 antibodies (SDC1: 1:50, CD34: 1:200) at 4°C overnight, and then counterstained with hematoxylin. Images were then obtained using an Olympus Bx51 optical microscope equipped with a Sony SLT-A77 digital camera. The Weidner's highest vessel density counting method was used to quantify microvessels in the specimens [39]. Microvessel counts were obtained by identifying areas with increased microvessel density (i.e. hot spots) under 40x magnification; microvessels were counted under 200x magnification. Microvessel density (MVD) was defined as the mean of the counts obtained for five of these fields.

### Database mining

SDC1 gene expression data for glioma samples were obtained from two publicly available databases: (i) TCGA, which combined low-grade glioma (LGG) and GBM datasets (https://cancergenome.nih.gov/) generated using Illumina HiSeq 2000 RNA Sequencing, and (ii) the NCBI-GEO GSE4290 dataset, derived using the Affymetrix HG U133 Plus 2 platform. Samples from epilepsy patients in the GSE4290 dataset were used as the negative control group. The expression data were analyzed using the survival package for R statistical software.

### Statistical analysis

Unless otherwise stated, results are reported as the means ± SD. All experiments were performed independently at least three times. Differences between groups were evaluated using either Student's *t*-test or ANOVA, and *p*<0.05 was considered statistically significant. SDC1 expression in glioma samples from the databases was compared using the Kruskal-Wallis non-parametric test. Survival curves were analyzed using log ranks and the Wilcoxon test. GraphPad Prism 5 (GraphPad Software, Inc., San Diego, CA, USA) was used for data analysis.
